# Degradation Mechanisms of Mortar and Plaster Layers

**DOI:** 10.3390/ma17143419

**Published:** 2024-07-11

**Authors:** Miloš Drdácký, Radek Ševčík, Dita Frankeová, Veronika Koudelková, Jaroslav Buzek, Marek Eisler, Jaroslav Valach

**Affiliations:** 1 Department Heritage Science, Institute of Theoretical and Applied Mechanics of the Czech Academy of Sciences, 190 00 Prague, Czech Republic; 2 Department Material Research, Institute of Theoretical and Applied Mechanics of the Czech Academy of Sciences, 190 00 Prague, Czech Republic; sevcik@itam.cas.cz (R.Š.);

**Keywords:** plaster buckling, mortar disintegration, salt crystallization effects, NDT topography, IEC salt analysis, failure analysis

## Abstract

This article presents a case of complex investigation of defects of lime mortar and plaster that have been developing over a period of 48 years in a house in Prague and are strongly influenced by thermal and salt crystallization cycles. The aim of this research was to describe the degradation phenomena of mortars and plasters observed on a narrowly limited part of the building, combining structural elements of different types and ages and to explain the mechanisms of their formation and development. The geometric characteristics of the defects were determined by non-destructive methods, especially optical interference moiré, laser profilometry, photogrammetry, and infrared thermography. Material data were determined on samples by electron microscopy, ion exchange chromatography, and direct moisture content measurements. The results supported the hypothesis of the increase in the deformation of large buckles of detached plasters by the mechanism of buckling caused by loading of the edges with compression generated by volume changes. Direct loading of the boundary surfaces causes the formation of bulges in the confined areas. This study shows the importance of failure analysis of real structures to gain knowledge about the behavior of structures and materials under long-term service conditions.

## 1. Introduction

Degradation of plaster layers is the most common cause of the need for repairs to historical masonry buildings, both listed and unprotected. The problem of determining the causes of degradation and choosing the appropriate tools for their study has been addressed by a number of researchers, and a significant amount of literature has published the results of the research. For example, a recently published selection of literature focuses on a review of articles documenting the study of environmental factors in the onset of degradation, namely the action of salts, pollution, and biological growth [1]. Mortars and plasters are considered quasi-brittle artificial composite materials with important similarities to natural stones, especially sandstone. Therefore, numerous studies take advantage of research into stone degradation processes and their manifestation, for which terminology glossaries have been developed. The most recent one, issued by ICOMOS and translated into several languages, defines observable phenomena on stone architectural elements or art objects without analyzing their causes or origin [2]. One of the authors of this paper suggested a similar glossary for defects of surface mortar layers [3,4], in which possible causes of the occurrence are indicated. The main focus was on the detachment of plaster from its substrate. Dealing with the causes and proposing repairs is provoked in particular by the fact that such plasters are the load-bearing layers of wall paintings and frescoes. The compilation work [5] summarizes five causal groups of the emergence of plaster detachment: (1) thermal dilation stress; (2) pressure from crystallization of salts; (3) moisture and humidity with hydric/hygric dilation stress or weakening and dissolution of materials; (4) differential shrinkage and hardening in multilayers systems; and (5) external forces and actions.

Interest in plaster detachment has also sparked increased interest in the development of measurement and monitoring methods to determine its extent and behavior, especially changes over time. Because monitoring is applied on plasters with works of art, non-destructive, non-contact methods are preferred [6]. For the identification of hidden defects, passive and active thermography has proven to be the most effective and affordable method [7,8,9], especially in its naturally (environmentally) excited active variant [10]. In the environmental excitation variant, differences between daytime and evening or night temperatures are exploited. For example, after a warm day, in mild climate areas, the outdoor temperature drops in the evening to values that are sufficient for natural cooling of the investigated surface. Subsurface defects have also been successfully localized by measurements of differential surface vibrations of detached plaster areas on the basis of the Doppler effect when the surface is excited by periodical acoustic pressure [11], by piezo-shakers [12], or by electrodynamic shakers [13]. Acoustic tracing in its direct or semi-automatic version is also promising [14]. A useful review of methods and tools suitable for inspecting exterior surfaces is provided in [15]. Most of the above methods are not suitable for long-term monitoring of the behavior of detached plaster, especially when it is necessary to measure very small changes. For this purpose, a direct measurement of the deformation of the bulge peak by a contact sensor with multiplication of the measured value by an optical lever was successfully conducted [16]. The sensor consists of a hinged mirror mounted on a spike that passes through the plaster and is anchored to the wall. The mirror tilts when the deformation of the bulge transmitted by the tip, which rests on the surface of the bulge, changes. The tilt of the mirror is recorded by the shift of the laser beam reflection track on a screen. At a distance of 5 m from the mirror to the screen, the sensitivity of the bulge deflection measurement was achieved in values of ±1.25 µm. This sensitivity made it possible to monitor the behavior of the detached plaster during repeated changes in temperature and humidity over short and long periods of time. Of course, it is also possible to use a sensor with electrical measurement and data recording if the system is powered by electricity.

The authors of the cited work demonstrated reversible dislocations of the bulge surface caused by changes in temperature and humidity in the plaster. At the same time, they stated that the observed irregular irreversible changes may have been caused by a gradual increase in the tear-off and by blocking the return of elastic deformation by loose plaster grains in the gap between the substrate and the detached layer. They also drew attention to the different behavior of convex and concave bulges, which may occur exceptionally. When shrinking due to cooling or drying of the detached layer, the top of the convex bulge approaches the base, while the opposite is true for the concave layer. In conclusion, they stated that the process of surface layer separation is the result of the interaction of seven various influences. Of them, the most important seem to be as follows: (a) inborn material and production imperfections; (b) the initial shrinkage of the layers during maturation and hardening, when early defects in cohesion with the substrate may already occur; (c) shrinkage and swelling in response to temperature and humidity changes; and (d) loose particles in the gaps between detached layers. That interesting work was based on the long-term research of real surface defects in the convent in Müstair, Switzerland.

In the research on plaster degradation, most attention is paid to the influence of water-soluble salts. Water is generally considered to be the greatest enemy of monuments, and porous materials are undoubtedly the most Threatened. Leaving aside aesthetic defects caused by surface salt crystallization, the greatest risk of salt-related degradation is salt crystallization in the pores, accompanied by volumetric expansion and the development of damaging stresses [17]. Salts can crystallize in many ways and create many forms [18]; even for only common ions in building materials, the list of salts reaches 70 items [19]. The damage mechanisms also depend on the nature of the pore system within the infested material [20]. However, when evaluating the damage to building materials caused by salts, the danger posed by crystallization pressures is still mostly stated, mostly without reliable direct quantification. Estimates range from units MPa to around 150 ± 50 MPa [21]. The value depends on the crystal growth conditions, the experimental approach, and the evaluation of the measured data, which is sensitive to the accuracy of determination of the size of the crystal contact area with its support [22]. This recent thesis provides high-accuracy data on the crystallization pressure measured with a special device. The highest pressure values, directly measured in the confined space, as well as the considerable damage of sandstone, were determined in experiments with a sodium sulfate solution. The pressure values were experimentally measured within the range of 7 and 23 MPa. The fundamental parameter that leads to rapid crystal growth and the generation of tension stress sufficiently high for the creation of damage in the porous material is the supersaturation of the solution [23]. Nevertheless, temperature and relative humidity also play an important role, particularly the periodic changes in the conditions inducing dissolution and recrystallization cycles. The dissolved ions of salts tend to migrate in the porous network and accumulate in the zone of enhanced evaporation in the material. Thus, the progressive filling of pore space over time leads to the contact of crystals and pore walls. The salt solution in real masonry most frequently contains a mixture of different ions. The type of salts that can crystallize from the solution is strongly affected by the temperature, relative humidity, and the availability of ions. The nature of salt crystals, particularly their solubility, is modified by the mixture composition and differs from the properties of crystals formed from a single solution. The types of real salt mixtures found in buildings are considerably variable, and the modeling of crystallization from such solutions has recently been an intensively studied problem [24].

This article presents a case of complex investigation of defects of lime mortar and plaster that have been developing over a period of 48 years in a house in Prague and are strongly influenced by thermal and salt crystallization cycles.

## 2. Materials and Methods

The presented situation investigated a full-scale, non-planned experiment located in a rebuilt family house in Prague. The original ground-floor house was built in the middle of the 19th century in a then-suburban Prague area called Troja. It had stone masonry walls of about 60 cm thickness, timber floor, and a saddle roof covered with ceramic tiles. The house was refurbished with an extension of the first floor and an attic in 1976, while a part of the original ground-floor masonry was preserved.

**Figure 1:**
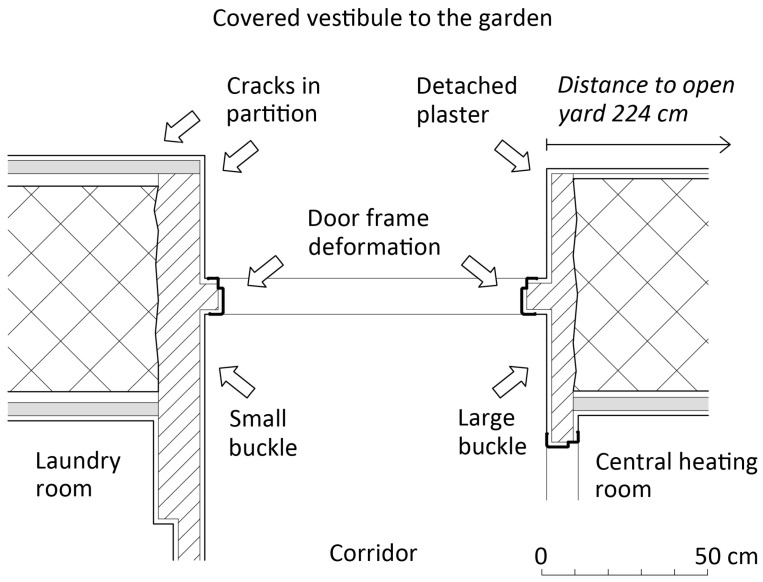
Situation of defects in the complex composite structure—stone walls (lattice hatching), recycled burned brick walls (diagonal hatching), and hollow partition brick lining (gray) built with an air gap between the lining and the stone wall.

The studied problem is located at the junction of the original stone argillite masonry and the new brick partitions (Figure 1). The whole detail is now located inside the layout of the house. In the past, the house ended with a stone wall and was connected to a yard, probably also used for breeding domestic animals—typically sheep in this area. The stone masonry was not insulated against rising damp, and during the reconstruction, the majority of interior surfaces were provided with 4 cm thin masonry partitions with ventilated air gaps. A door opening was cut into the stone wall, and its lining was fixed with a brick cladding without an air gap, i.e., in irregular contact with the ancient stone masonry. A thin-walled steel door frame was fitted for the door. The knot of walls around this door creates several specific conditions conducive to the gradual degradation of the plasters, as clearly shown in Figure 1.

In addition to the technical treatment of the surfaces of the historical stone wall, mortars of various quality are also used here, and the surfaces are adjacent to an environment with different moisture and temperature parameters. (For example, in cool April 2024, the vestibule T was 12.1 °C, RH 44%, corridor T 15.7 °C, RH 41%, laundry T 14.8 °C, RH 43%, and the central heating T 20.5 °C, RH 39%). The vestibule to the garden on the east has a large glass wall and door toward the south, which enables intensive insolation during winter months. The corridor area inside the house, as well as the laundry room, have a rather balanced climate throughout the year without dramatic changes in temperature and humidity. The room with central heating is about 5° to 10 °C warmer during the winter than the other spaces.

**Figure 2:**
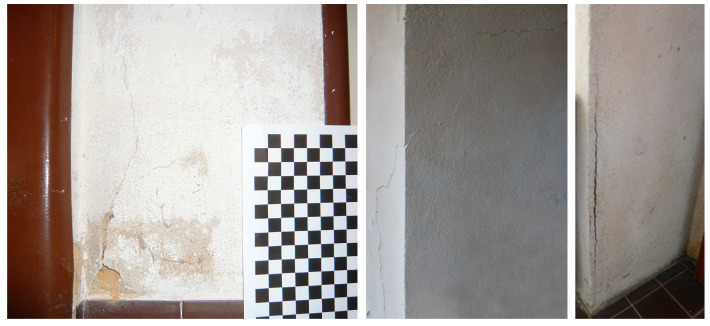
Plaster buckle with severe disintegration of mortar developing—condition in 2014 (**left**). Cracks around the northern corner and the southern corner in the vestibule (**right**).

This complexity of conditions was also reflected in the degradation patterns of the surrounding structures, which arose during the use of the building. In the corridor area, the most striking was the defect of the plaster on the lining of the door, which detached and created a large buckle with its extent continuously growing (Figure 2).

On the opposite wall, a significantly smaller buckle had developed. The steel door frame was also deformed, and the bowing deflection increased as well. These defects are described in detail below. The cracks around the northern corner of the vestibule are apparently of a different nature and can be attributed to uneven thermal expansion of the composite structure. This is also true for the southern corner, where the action of salts cannot be excluded either (Figure 2).

**Figure 3:**
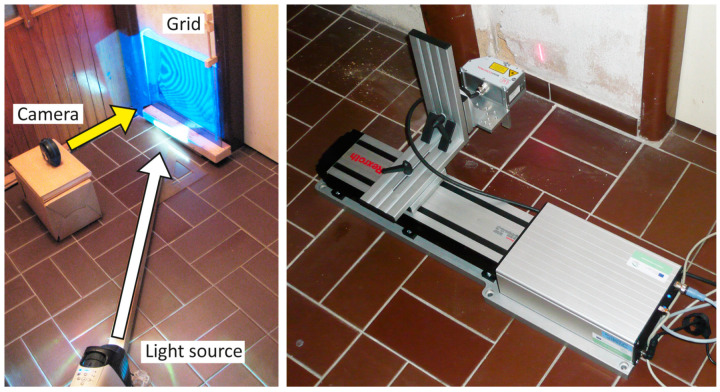
Moiré measurement set-up (**left**). Profilometry measurements (**right**).

For the measurement of the buckles’ geometry, contactless methods were used. The plaster buckle was documented using shadow moiré, which utilizes the interference of a grid projected onto the deformed surface and a non-deformed grid. The curved shadow lines on the buckle interfere with straight lines of the grid and form typical patterns, referred to as “moiré”. In a special geometrical arrangement of the measured surface, the grid, the projector, and the observer (camera), the generated moiré curves correspond to places equal in distance from a reference plane and represent contour lines of the mapped surface. The experimental set-up is schematically shown in Figure 3; a 1 mm grid was used for the measurements.

The geometry of the buckles was further analyzed by means of laser profilometry (Figure 3). This method utilizes a distance-measuring optical sensor based on the triangulation principle. The optical sensor enables simultaneous measurement of approximately a thousand positions in a band with a width of about four centimeters. Once attached to a translation stage, the length of the measured band can reach up to approximately 30 cm. The result of the measurement is a dense cloud of surface points separated by tens of microns, with a height resolution of a few micrometers. Profilometry was also applied in the door frame buckle measurements. The data here were checked with contact measurement using a caliper supported by a massive straight double rail and with a contour gauge profile tool.

Photogrammetry was further used to create a 3D model of the complex shape of the thin-walled steel door frame. The process consists of capturing photographs from multiple positions and their subsequent processing using computer software. For the presented case, Agisoft’s Metashape was applied.

The extent of the plaster detachment was measured using thermal imaging under a forced temperature gradient, which was generated by brief heating of the air in the limited space of the corridor. Then, the sequence of change of thermal patterns was registered during the cooling of the wall after the removal of the heater. The acquired thermograms were searched for the areas changing temperature at a different rate than their surroundings—anomalies that may indicate the location of air pockets resulting from lack of adhesion. The extent of the detached plaster was further checked by acoustic tracing, which consists of the identification of changes in the sound emitted by the surface when tapped.

The moisture content in the surface layers of the masonry was measured using the high-frequency microwave sensor Moist 210 B produced by HF sensor GmbH, Leipzig, Germany. Plaster index measurements were calibrated using an extracted bulk of material subjected to a drying and weighing procedure. In both cases, the MOIST R1 measuring probe was used for surface layers to a material volume depth of 2–3 cm.

**Figure 4:**
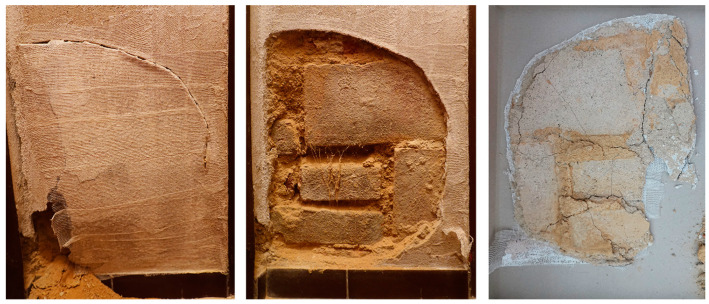
Sequence of plaster sample extraction (fixing the detached plaster—**left**, the wall surface after plaster removal—**center**, and the reverse of the removed layer of plaster—**right**).

For the material analysis, a destructive approach was adopted. The bulging plaster was removed from the wall after fixing it with a thin cloth and Metylan restoration glue, similar to the procedure for plaster transferring. The buckled plaster was cut off along its edge (Figure 4), and the obtained plaster plate was used for material investigations.

For the SEM analysis, four samples were collected from different positions of the tested body. The prepared polished sections and fractured pieces were gold-coated prior to the observations under a scanning electron microscope (SEM, Quanta 450 FEG (FEI, Brno, Czech Republic)) to reduce charging effects. SEM images were collected using the Everhart–Thornley detector.

For the IEC (ion exchange chromatography) analysis, the collected samples were dried to a constant weight. Aqueous extracts were prepared from the dried and ground samples (sample weights and solution volumes are provided in Table 1 and Table 2). After 60 min of stirring at laboratory temperature on a magnetic stirrer, the solutions were allowed to stand for 24 h and then filtered (Munktell, Falun, Sweden; 87 g m^−2^) into 100 mL volumetric flasks. The undissolved portion was washed with approximately 5 mL of deionized water, and after filtering, the volumetric flasks were refilled to full volume. Water-soluble cations and anions in prepared extracts were determined on an ion exchange chromatography (IEC) Dionex ISC-5000 (Thermo Fisher Scientific, Waltham, MA, USA) using mobile phases of a mixture of 4.5 mM carbonate with a flow rate of 1.2 mL min^−1^ and 1.4 mM bicarbonate and 20 mM methanesulfonic acid with a flow rate of 1.0 mL min^−1^, respectively. The signal was detected using a conductivity detector.

An X-ray powder diffraction (XRPD) pattern of the ground mortar was collected using a Bruker D8 Advance diffractometer (Billerica, MA, USA) with the following parameters: Cu Kα radiation (λ = 1.5418 Å), voltage 40 kV, and current 40 mA. The pattern was measured in the angular range 5–80° 2θ, counting 0.320 s for each step of 0.012° 2θ. Quantitative phase analysis was performed with Rietveld analysis using Topas 4.2 (Bruker AXS) software.

Energy-dispersive X-ray spectroscopy (EDS) was performed using an EDAX (Ametek, Berwyn, PA, USA) detector. The collected spectra were evaluated in Team V3.2 software.

## 3. Results

### 3.1. Geometrical Data

**Figure 5:**
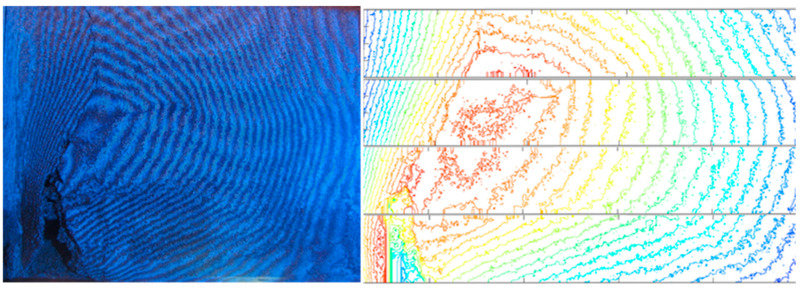
Moiré topography contour lines with 1 mm spacing of the buckle of detached plaster (**left**); visualization of profilometry measurements (**right**). (The studied area is shown in Figure 3).

The large buckle of the plaster reached its peak of about 26 mm after 48 years, as documented in Figure 5. It is clearly seen that the deformation in the buckle exceeded the elastic limit of the mortar material, and linear hinges developed.

**Figure 6:**
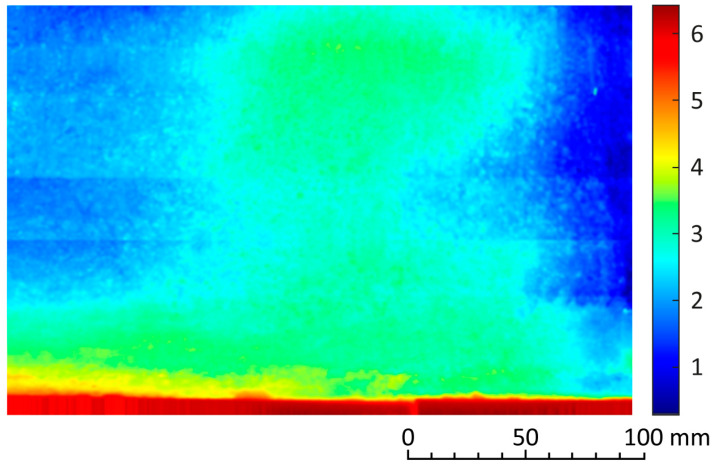
A color-coded deflection map of surface points expressed as a distance (in millimeters) from the vertical plane set as the zero value, outlining the size and shape of the detachment.

**Figure 7:**
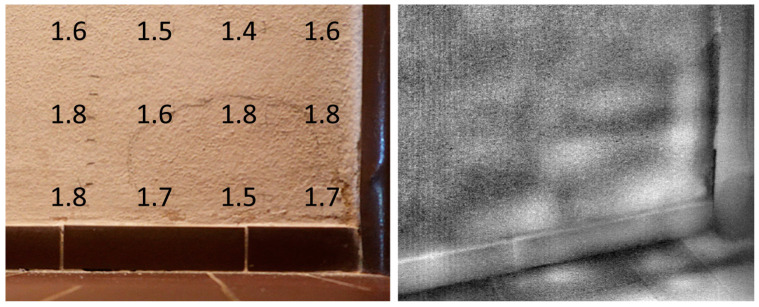
Extent of detached plaster identified by acoustic tracing with the measured moisture content w_m_ (wt.%) in the plaster (**left**) and thermography (**right**). The lighter areas in the thermogram roughly correspond to the detached part of the plaster, which should increase in temperature slightly faster during heating of the room.

The small buckle on the opposite wall, described with data from the profilometry, thermography, and acoustic tracing, has a comparably negligible extent and a detachment with a peak of only 3 mm (Figure 6 and Figure 7).

**Figure 8:**
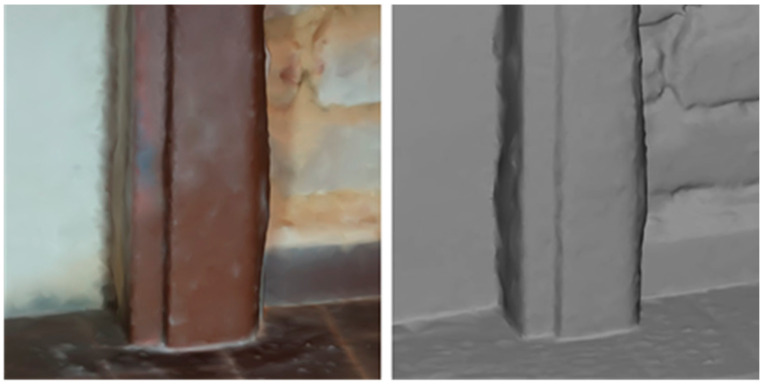
Photogrammetric reconstructions of the deformed door frame.

**Figure 9:**
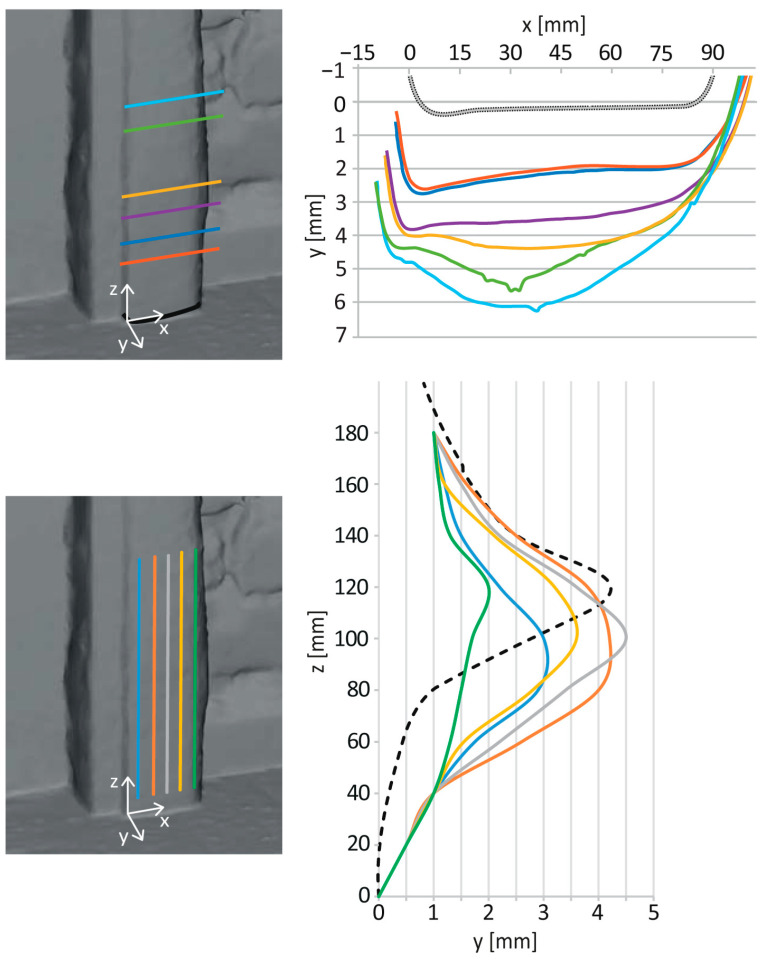
Color-coded profiles of the deformed steel door frame post adjacent to the large buckle—transversal (**top**), vertical (**bottom**). The dashed line in the bottom right graph presents the central vertical deformation of the left door post close to the smaller bulge.

The thin-walled steel frame of the door is strongly deformed in the part adjacent to the floor. The complex deformation shape of the right pillar is shown in the photogrammetric model in Figure 8. A detailed profilometry measurement of the transverse profile at levels distanced 30, 55, 75, 95, 120, and 140 mm above the floor is shown in Figure 9. It also presents the course of vertical deformation of the frame in five vertical profiles 10, 25, 35, 50, and 60 mm distant from the door rabbet stop, as well as the deformation of the left post of the door frame in the central vertical profile.

### 3.2. Moisture in the Walls

Three batches of material were taken from the vicinity of the removed plaster bulge for moisture analysis and subsequent calibration of microwave measurements. The humidity of the *w_m_* was determined by weighing the samples after delivery to the laboratory and after drying it to a steady weight of 105 ± 5 °C according to the equation:

(1) $$w_{m}=\frac{m_{s}-m_{d}}{m_{d}}\times 100$$

*w_m_*—moisture by weight (wt.%);
*m_s_*—mass of the element in the state after removal (g);
*m_d_*—the weight of the element in the dried state (g).

The results are summarized in Table 1.

**Figure 10:**
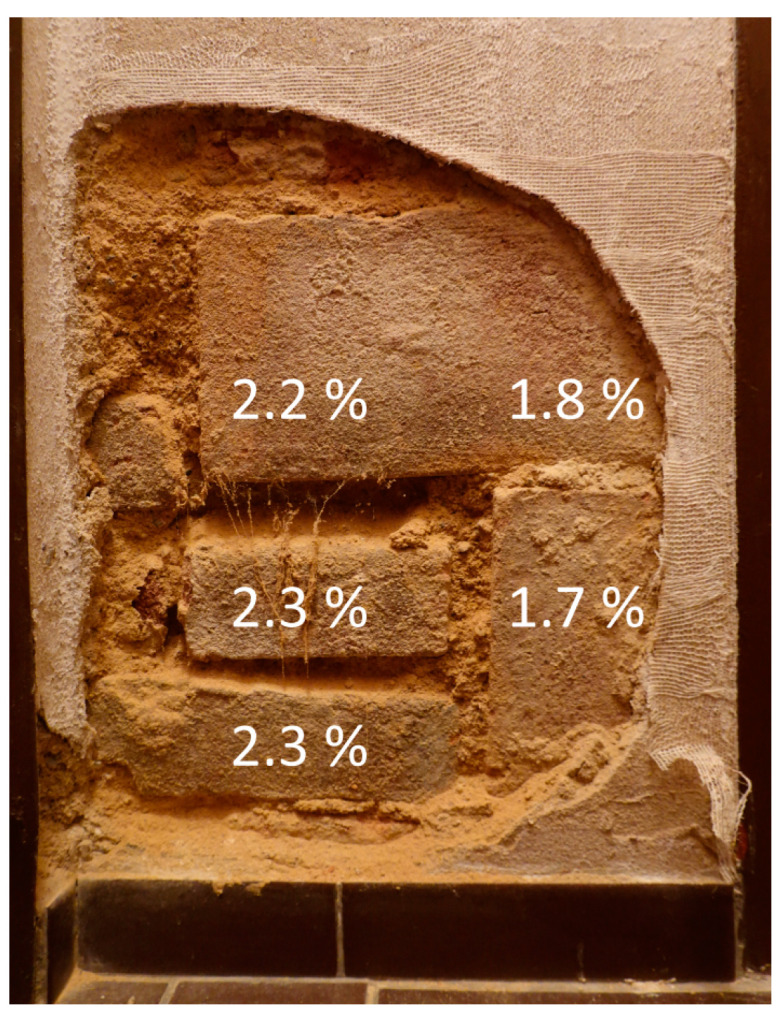
Map of moisture content in % of weight in the bricks in contact with the ancient masonry wall where the large buckle has developed over decades.

The moisture indices obtained by microwave measurements can be converted into the percentage moisture content of the plaster using the values from Table 1. The measured values of the area around the faint bulge showed only slightly higher humidity compared to the detached part (see Figure 7). The directly measured moisture in the bricks of the opposite wall where the large buckle developed revealed a higher water content (Figure 10). The moisture in the surface layers of the other walls ranged between 1.2% and 2%. Microwave measurements are influenced considerably by the roughness and deviations from the flatness of the surface; therefore, they are only approximate estimates of moisture content, which nevertheless provide a picture of the relative situation in a larger context and show that the moisture content in the plaster layers of the walls is rather low.

### 3.3. Material Analysis

**Figure 11:**
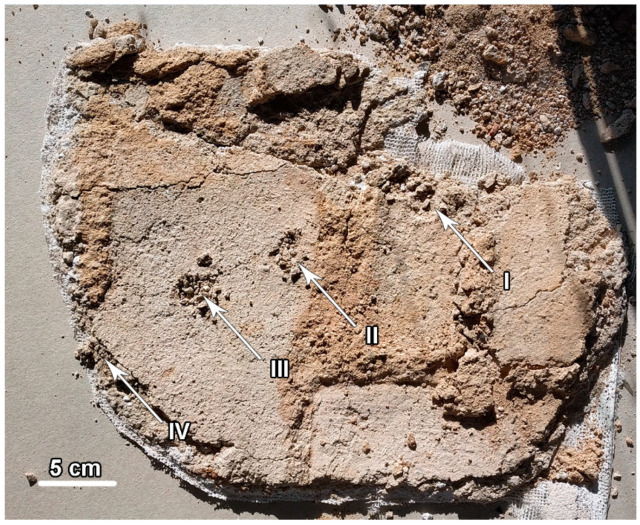
Sampling points for the IEC and SEM analysis.

Tests for the presence of salts and microscopic analyses of the structure were carried out on the removed floe of plaster layers. The sampling points for analyses are marked in Figure 11.

The results of the quantitative determination of anions and cations from aqueous extracts are presented in Table 2 and Table 3, respectively.

The results from the ion chromatograph measurements showed that of the salts, the highest concentrations were detected for nitrates, which corresponds to the historical exploitation of the yard for breeding domestic animals.

**Figure 12:**
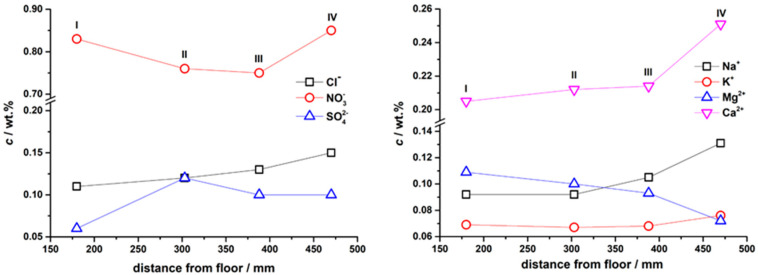
Anions and cations distribution in the extracted detached plaster floe.

In the ion analyses, the Cl^−^ showed an increasing concentration in the vertical direction of the wall. The same goes for Na^+^ and Ca^2+^ ions. Interestingly, the trend is the opposite for Mg^2+^. Others do not show a clear dependence. The results are graphically presented in Figure 12.

The plaster of the wall was made of slaked aerial lime and local sand as a two-layer structure of a core and a thin stucco layer on the surface. The composition of the mixture is unknown, but on similar constructions, masons also added a small amount of cement to the lime mortar. However, it was a mortar of lower strength and unable to successfully resist the repeated action of subsurface crystallization of salts.

**Figure 13:**
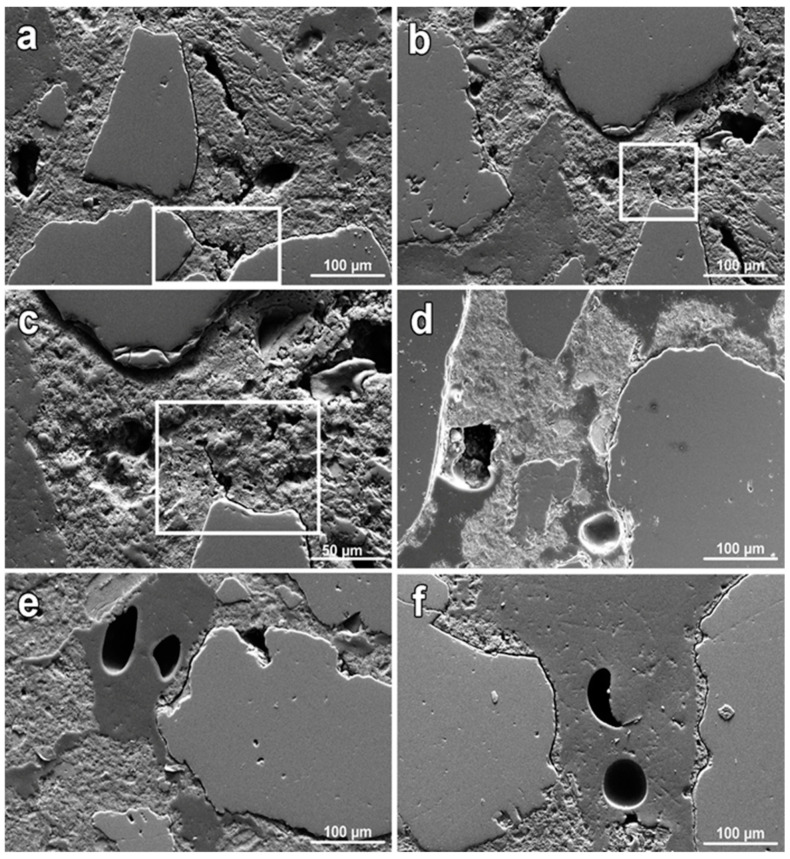
SEM images of prepared mortar sections taken at 800× magnification ((**c**)—1600×): (**a**)—sample I, (**b**)—sample I, (**c**)—crack detail (sample I), (**d**)—sample II, (**e**)—sample III, (**f**)—sample IV. Cracks in sample I between the two grains (**a**) and the crack advancing into the binder (**b**,**c**) are highlighted by white rectangles.

For electron microscopy, collections of cross-sections were prepared and examined at 100× and 800× (Figure 13) magnification. At higher magnifications, a gap between some grains and the binder was detected in all samples. In sample I (taken in the most degraded section), cracks between the grain–binder–grain system were also detected (Figure 13a), as well as a crack propagating from the grain to the binder (Figure 13b,c).

XRPD measurements unveiled the composition of the mortar. It was found to be a mixture of quartz (SiO_2_—75.6(5) wt.%), calcite (CaCO_3_—17.3(3) wt.%), microcline (KAlSi_3_O_8_—3.0(3) wt.%), muscovite (KAl_2_(AlSi_3_O_10_)(F,OH)_2_—4.0(3) wt.%), and traces of rutile (TiO_2_—0.1(2) wt.%).

In accordance with the XRPD measurements, the EDS mapping showed that the mortar sample contained Si, Ca, Al, O, Mg, K, Cl, and C elements. The aggregate grains are composed solely of silicon oxide or in combination with aluminum and potassium ions, confirming the presence of quartz, feldspars (microcline), or phyllosilicate (muscovite). The binder is mainly composed of calcium, oxygen, and carbon. In addition, a small amount of Cl was detected in the measured area.

**Figure 14:**
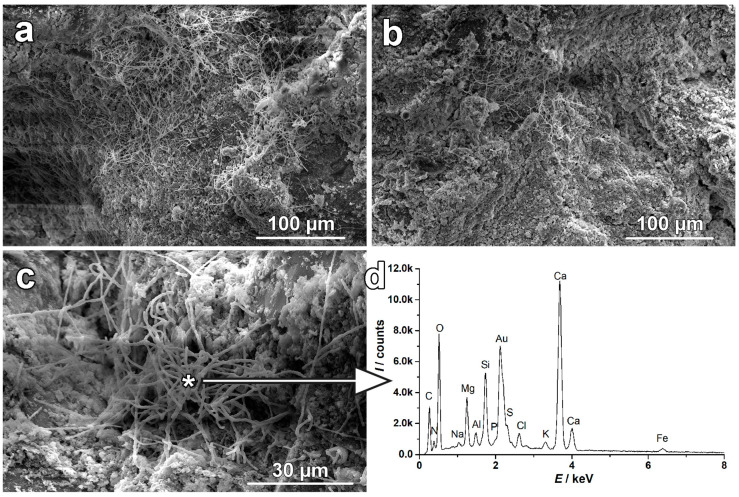
Collection of SEM images of a small localized family of fibrous features on the surface of plaster (**a**,**b**), their morphology observed at higher magnification (**c**), and EDS elemental spectra (**d**) analyzed in fibers marked with an asterisk.

Microscopic examination of the surface of the detached plaster showed small isolated areas with a high concentration of fibrous formations, as depicted in Figure 14. These fibrous features, mostly presented as complex clusters, had a width of around 1 µm with variable lengths from a few micrometers to tens of µm. Based on their morphology, such features could be ascribed to the crystals of inorganic salts [25] formed due to antitaxial growth during desiccation [26]. Filament nests are not interconnected and do not form the coating that is typical of biological films of fungi residues [27]. EDS spectra indicate that these are crystals of a mixed salt.

## 4. Discussion

Several decades of monitoring the development of plaster failure in the interior of a building by the action of salts is a unique experiment not yet described in the literature. The special situation with random conditions of action allowed for multi-parametric analysis of the observed disturbances or results and provided generally applicable knowledge.

The studied defects represent three different situations. The large bulge on the detached plaster is in contact with masonry, which is a source of rising damp and soluble salts only on its perimeter, and this was probably the case throughout its development. Thus, the bulge enlargement must be attributed only to the stability buckling phenomenon caused by compressive internal forces along the boundary. In this case, compressive forces cannot be induced by external mechanical loads. They are undoubtedly generated by the volumetric expansion of the material. There are two possible sources of such expansion—the impact of thermal and hydric/hygric dilation and salt crystallization with subsequent microscopic disintegration of the material. Thermal and hygric/hydric dilation generate mostly reversible volumetric change even though some irreversible components might be present if the dilation cycles cause internal damage to the original compact composite structure, which is typical after multiple repetitions. The salt crystallization causes the initiation and propagation of micro-cracks, which destroy compactness. In the studied case, the boundary conditions severely affect the resulting defect. The corner border of the initial detachment adjacent to the bottom and door frame lines are constrained in their development. The other parts of the bulge border shift with each crystallization cycle at the weakened area of contact between the plaster and the wall. Therefore, the damaged strip of plaster is localized, and the detached free area of the buckle is less affected. The above-mentioned corner is more damaged due to permanent saturation and crystallization throughout the whole bulk of the mortar, which ultimately results in complete disintegration. The hypothesis of the buckling phenomenon generated and supported by the volume increase due to material disintegration corresponds with the results of a study on thin plaster layers detached from the Valtice Chateau façade, which were discovered to have a high degree of microscopic grain–binder separation [28].

In principle, the same mechanism should be manifested on the opposite wall, where, however, over the same period of almost 50 years, only an indistinct bulge of torn plaster has formed. This is probably due to a very different situation regarding the transport of moisture with water-soluble salts, as well as a significantly different temperature regime in the masonry, especially in the cavity beneath the detached plaster. Here, the plaster was probably torn off solely due to the action of salt crystallization, and the phenomenon of buckling did not develop. The wall area is not exposed to significant changes in temperature, humidity, and evaporation cycles.

The observation of the localized occurrence of fibrous crystallization habits on the detached plaster of the large bulge corresponds to the conclusions of the already-mentioned work [26]. The occurrence of these spots is rather rare and formed in places of a very thin layer of salt solution on the interior surface of the detached plaster. Salt whiskers were identified as frequent salt crystallization habits damaging bituminous roads [29]. Damage most likely occurred where there were large diurnal changes in temperature and relative humidity and where evaporation is generally high and cyclic. Such situations could occasionally be present in the gap between the detached plaster and the wall adjacent to the heated space in the investigated case. Some scientists observed similar formations of halite, as discussed in the literature [30], and the authors have experienced an example of natrium nitrate. Stresses and related damage that can induce crystallizing nitrates are significantly high since the salt solution can achieve considerable supersaturation during evaporation or cooling experiments. Such results were presented in a recent study [31]. Damage was observed in the examined specimens even with moderate salt content (below 1.5 wt.%).

The bulge of the door frame represents the third situation. Here, the steel frame functions as a partial confinement for the mortar layer, and the bulge develops as a bow due to the pressure of the material accumulated between the door frame and the wall. Undoubtedly, the pressure here is also increased by the contribution of corrosion products to the increase in the volume of material behind the steel frame. The deformation of the door frame is approximately the same in the right and left posts, where the maximum deflection of the thin-walled profile reaches a size of between 4 and 5 mm, with the peaks lying at a distance of 100–120 mm from the floor. The protrusion of the peak at a greater height above the floor can be explained by the fact that there is not such a massive crystallization of salts near the floor due to the reduced possibility of drying. The steel frame is torn away from the masonry, and the resulting gap allows crystallization to occur from an approximate level of 50 mm above the floor. However, even here, the right side is more deformed, probably due to the above-mentioned conditions of crystallization cycles.

The measurement of the shape and rise of the bulges of the detached plaster using the moiré method can be evaluated as very fast and sufficiently accurate. In addition to a camera and a light source, e.g., a slide projector, a glass plate with an even grid of lines is needed for implementation, which is not a common piece of equipment. However, the acquisition of a line grid is not expensive. The advantage is that with a suitable arrangement of the experiment, we obtain an image of the contour lines of the surface immediately without the need for further processing.

Usable results were also obtained by applying a laser profilometer. Here, in order to create the topography of the deformed surface, we cannot do without further data processing, which requires some time. A profilometer is an expensive instrument and is rather suitable for laboratory work. In the field, it needs the support of a solid scaffolding. The results confirm that a simple topography of an indistinct bulge on the plaster does not sufficiently identify the extent of the plaster detachment and must be verified by another method, e.g., acoustic tracing.

Although the authors of this article have very suitable experience with the use of thermography for the detection of subsurface defects, in this case of the very flat bulge, the measurement was not sufficiently contrasting. This is probably due to the fact that the slight detachment still has the cavity partially filled with fallen grains of plaster or salt crystals. In the case of unclear detachment boundaries, the extent of the defect had to be confirmed by the classical method of acoustic tracing.

The photogrammetrically created 3D model allows for studying the mutual relations of the deformation of more complex building nodes. In this case, it helps to evaluate the extent of the deformation damage to the thin-walled door frame.

The knowledge gained from the analysis of the studied faults is useful for considering measures that can mitigate or eliminate them in historical buildings. An essential factor is the reduction in or prevention of the possibility of salt crystallization within the structure. This can be achieved in several ways, which we present below without details, beyond the scope and intent of this article. It is important to reduce the mobilization of water-soluble salts. Water-soluble salts are present in the masonry of most historical buildings or in their subsoil, and therefore, it is necessary to protect these elements and areas from the supply of water. Perfect drainage of rainwater from the building is essential. It is effective to insert mechanical or chemical barriers against the capillary rise of water and salt solution into the masonry, which, however, may not always be usable or acceptable in historical masonry. Another option is to reduce the salt content or its concentration in the masonry by applying some of the desalination methods, e.g., clay or cellulose-based poultices, electromigration and vacuum, or pressure water extraction. Crystallization inhibitors are also studied, which either affect crystallization pressures or ensure the formation of salt crystals on the surface instead of inside the porous mortar system. When replacing completely degraded plasters that can no longer be salvaged, it is useful to carry out desalination before applying new plaster and, if possible, to use sacrificial plasters with controlled capillarity and increased resistance to disintegration.

The detached plaster layer can be re-attached to the masonry by grouting the cavity. Such a procedure would probably not be successful for both detachments presented in this article because the indistinct cavity is probably filled with components of the salt-disintegrated mortar, and the larger cavity is too bulky. If a large plaster bulge carries valuable information on the surface, then it may be recommended to remove it from the wall in the way that was used in our case and, after flattening and consolidating, fix it again to the masonry. However, the growth of the defect by the buckling mechanism is slow, and the danger of a critical situation can be detected by careful monitoring with measurement of the growth rate by any of the presented methods. The method of preventive intervention must then be decided together with the restorers.

## 5. Conclusions

This article shows the importance of a scientific approach to the analysis of defects and failures observed during surveys of the condition of historical buildings. In practice, dilapidated plaster layers are often removed without a detailed consideration of the reason for their degradation. The appearance and development of defects in plasters attract attention only if they are carriers of valuable information, especially artistic or decorative works. However, such plasters usually do not allow for destructive tests to be carried out. The results presented in this article therefore support the development of a broader interest in the behavior of plasters and the use of the possibilities of studying their behavior in cases where they are removed and thus provide material for deeper study.

The investigated degradation depends on the local situation, the materials used, and their mutual time-dependent interaction in the conditions of a real random process of accumulation of initial conditions and long-term cyclical environmental influences. It was an unplanned experiment for the evaluation of which modern methods of material analysis and inverse reconstruction of defect formation were applied.

The unique novelty of this study is its coverage of a long-term decay process gradually developing from degradation to failure. The time span of the process covered exceeds the duration of a typical research project by multiples and therefore remains usually “invisible” for scientific investigation. The duration of the studied degradation process helped to reveal the mechanism leading to the development of defects that arise only under certain conditions. However, such a detailed analysis of plaster is not always relevant or economically viable for every repair or restoration of historical buildings.

The collection and analysis of real situations of defects and failures observed on buildings and materials is a valuable source of knowledge even though such non-planned experiments do not provide us with complete and systemic sets of data. Failure analysis is still a discipline that struggles to attain an adequate position in research. This is due to a number of causes, the most important of which is the reluctance to publish failures in technical solutions that their creators consider personal failures. However, consistent detection of the causes and mechanisms of failures and their disclosure is the only effective way to reduce the occurrence of critical failures and the resulting damage. Failure analysis is an inspiring source of further research, as it often ends up with questions that have only been answered with a certain degree of uncertainty, as in this case.

## Figures and Tables

**Table 1:** Moisture content by gravimetry.

Sample	$m_{s}$ (g)	$m_{d}$ (g)	Water Content (g)	$w_{m}$ (wt.%)
1	62.381	61.219	1.162	1.9
2	68.808	67.586	1.222	1.8
3	59.061	58.252	0.809	1.4

**Table 2:** Quantitative determination of anions from aqueous extracts.

Sample	Weight/g	c/mg L^−1^			c/wt.%		
		Cl^−^	NO_3_^−^	SO_4_^2−^	Cl^−^	NO_3_^−^	SO_4_^2−^
I	0.499	10.82	82.68	6.18	0.11	0.83	0.06
II	0.547	12.91	83.52	13.29	0.12	0.76	0.12
III	0.553	14.64	82.49	11.53	0.13	0.75	0.10
IV	0.527	15.82	89.06	11.04	0.15	0.85	0.10

**Table 3:** Quantitative determination of cations from aqueous extracts.

S.	Weight/g	c/mg L^−1^				c/wt.%			
		Na^+^	K^+^	Mg^2+^	Ca^2+^	Na^+^	K^+^	Mg^2+^	Ca^2+^
I	0.499	9.17	6.86	10.90	20.48	0.092	0.069	0.109	0.205
II	0.547	10.03	7.38	10.92	23.23	0.092	0.067	0.100	0.212
III	0.553	11.65	7.48	10.24	23.64	0.105	0.068	0.093	0.214
IV	0.527	13.75	8.04	7.54	26.45	0.131	0.076	0.072	0.251

## Data Availability

The research data are available upon request from the authors.
